# Now and the Future: Medications Changing the Landscape of Cardiovascular Disease and Heart Failure Management

**DOI:** 10.3390/jcm14113948

**Published:** 2025-06-03

**Authors:** Thomas Oswald, Steven Coombs, Susan Ellery, Alexander Liu

**Affiliations:** Sussex Cardiac Centre, Royal Sussex County Hospital, Brighton BN2 5BE, UK; thomas.oswald@nhs.net (T.O.); steven.coombs@nhs.net (S.C.); susan.ellery1@nhs.net (S.E.)

**Keywords:** cardiovascular diseases, heart failure, SGLT2 inhibitors, GLP-1 receptor agonists, non-steroidal mineralocorticoid receptor antagonists

## Abstract

Cardiovascular diseases (CVDs) remain the leading cause of morbidity and mortality worldwide. Epidemiological data demonstrate that the overlap between CVD, Type 2 Diabetes (T2DM), chronic kidney disease (CKD) and heart failure (HF) is becoming increasingly apparent, with aging populations making these patient cohorts more difficult to treat. In the last decade, three standout drug classes have emerged with the potential to broaden the treatment options for patients with multi-morbid CVD and heart failure. These are sodium–glucose cotransporter 2 (SGLT2) inhibitors, non-steroidal mineralocorticoid receptor antagonists (MRAs), e.g., Finerenone, and glucagon-like peptide 1 receptor agonists (GLP-1RAs). These medications are now entering UK and European guidelines for the treatment of CVDs including HF whilst crucially providing associated prognostic benefits for patients with T2DM and CKD. The future of these agents for CVD risk stratification may involve primary care at the forefront, alongside tailored, patient-specific medication regimens. This review article aims to discuss these three main drug classes (SGLT2 inhibitors, GLP-1RAs and non-steroidal MRAs) in detail by exploring their current evidence base across heart failure (HF) and CVD management and future clinical implications of their usage as mainstream medical therapies.

## 1. Introduction

Cardiovascular diseases (CVDs) are responsible for over 40% of deaths in Europe each year, and each day, 10,000 patients succumb to CVDs [1]. The continued lethality of CVD as a disease entity is undoubtedly exacerbated by the presence of co-morbidities such as Type 2 Diabetes Mellitus (T2DM) and obesity [2]. It is currently predicted that by 2050, more than 1.31 billion people worldwide will suffer from T2DM [2]. Evidence demonstrates that the primary reason for this is worsening obesity, a global health epidemic in and of itself, with current estimates of more than 3 billion people worldwide classed as overweight or obese in 2024 [3]. Indeed, this increasing prevalence of T2DM and obesity will further worsen their deleterious effects on CVD patients [4]. The link between obesity and heart failure with preserved ejection fraction (HFpEF) is now well-established, and the prevalence of HFpEF surges in parallel with the ever-worsening obesity epidemic [5].

**Figure 1:**
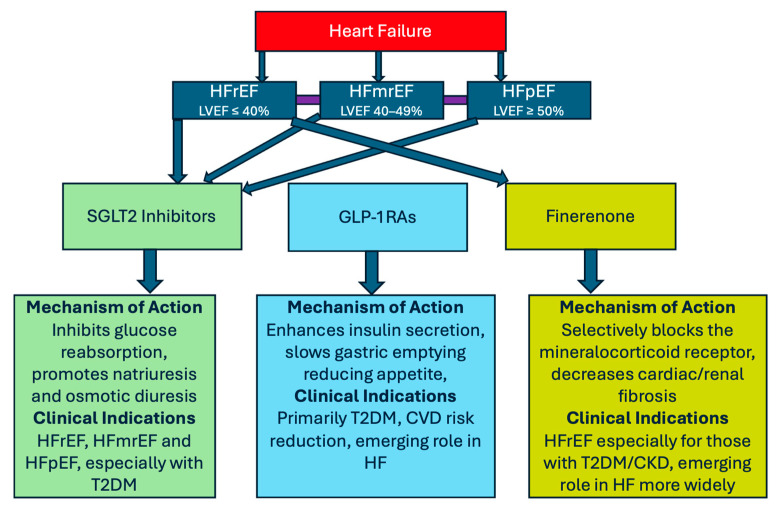
Visual schematic of the use of SGLT2 inhibitors, GLP-1RAs and Finerenone across different heart phenotypes with mechanisms of action and specific clinical indications.

Beyond the development in understanding of the power of current guideline-directed medical therapy (GDMT), in the last decade, novel anti-diabetic agents have emerged, which also demonstrate significant reductions in major adverse cardiac events (MACEs) including long-term cardiovascular mortality [6]. These prognostic benefits appear above and beyond those gained from other established therapies, such as high-dose statins and anti-platelet and anti-hypertensive agents [7]. Two well-known anti-diabetic agents with cardiovascular benefits are SGLT2 inhibitors and glucagon-like peptide 1 receptor agonists (GLP-1RAs). Additionally, Finerenone, a non-steroidal MRA, has recently surfaced from the already well-established class of steroidal-based MRAs that are used to treat patients with heart failure with reduced ejection fraction (HFrEF). Finerenone has been associated with a reduction in MACEs in patients with HFpEF and heart failure with mildly reduced ejection fraction (HFmrEF) [8]. The use of these medications across the spectrum of HF phenotypes is visually represented in Figure 1.

As medical therapy in patients with heart failure needs to be commenced early and continued with good compliance to both reach and maintain their optimal therapeutic effects, the introduction of novel therapies that add to our existing armamentarium and the expansion in the applicability of current GDMT to a broader spectrum of HF patients are exciting. Despite advances in our knowledge and treatment options for patients with HFrEF over the last 40 years, prognostic therapies for HFpEF remain limited. However, HFpEF patients continue to contribute to a significant proportion of all patients with HF. As we move into the next 20 years, effective development of novel therapies for HFpEF undoubtedly remains a research and clinical priority. This narrative review article focuses on the major clinical trials and studies in the last 10 years on heart failure management. It will examine three such novel medical therapies, their existing clinical evidence, and their potential clinical implications in the future.

## 2. Current Evidence for GDMT Across the Spectrum of Heart Failure

Current European Society of Cardiology (ESC) guidelines offer a class 1A recommendation for the use of “four pillar” medical therapy for patients with heart failure with reduced ejection fraction (HFrEF) [9]. This guideline-directed medical therapy (GDMT) consists of a combination of an angiotensin receptor blocker (ARB) with a neprilysin inhibitor termed sacubitril/valsartan, steroidal-based mineralocorticoid receptor antagonists (MRAs) such as spironolactone and eplerenone, beta-blockers and, more recently, sodium–glucose cotransporter 2 (SGLT2) inhibitors including dapagliflozin and empagliflozin [9]. Even in the recent past, most of the large trials that studied these medications focused solely on patient groups with HFrEF excluding large numbers of patients with symptomatic HF that did not fall into the category of having a left ventricular ejection fraction (LVEF) on transthoracic echocardiogram of <40% [10]. Following a greater understanding in the last decade of the ever-increasing patient population groups of heart failure with mildly reduced ejection fraction (HFmrEF) and HFpEF, which continue to expand alongside the worsening obesity crisis, there has been a push to discover new medications that offer prognostic benefit and explore the idea that current GDMT for HFrEF may extend to these subgroups.

Amongst the medication classes mentioned, evidence exists that supports the above notion of improved outcomes for these patients with current GDMT. Pooled data on post hoc analysis from the PARAGON-HF and PARADIGM-HF trials, which looked at sacubitril/valsartan vs. ARB for patient cohorts across the entire range of LVEF, showed the superiority of sacubitril/valsartan on rates of CV death and HF hospitalization with a significant benefit in HFrEF patients [11]. However, attenuation of this treatment effect only manifested in patient groups with an LVEF of >55% suggesting that in the range of 40–55% there may well be a prognostic benefit of using this medication class [11].

The multinational trial TOPCAT studied the benefits of the traditional MRA, spironolactone, vs. placebo in patients with an LVEF > 45%, with primary outcomes of HF hospitalization, aborted cardiac arrest and composite death from CVD causes, which demonstrated no statistically significant difference [12]. However, post hoc analyses of the regional data from this trial showed a marked four-fold difference in placebo event rates between patient groups from the Americas and Russia/Georgia [13]. On exclusion of the patients from Russia and Georgia, an 18% risk reduction was observed in the primary outcome of HF hospitalization and rate of CV death [13], implying potential benefit for traditional MRAs in patients with an LVEF > 45%.

Moreover, meta-analysis of 11 trials that focused on the use of beta blockers vs. placebo across the spectrum of LVEF demonstrated that for patients in sinus rhythm with an LVEF of between 40 and 49%, similar prognostic benefits in reducing all-cause and cardiovascular mortality were observed as in patient groups with an LVEF of <40% [14]. This result, however, was not mirrored in patients with atrial fibrillation (AF) [14].

Overall, these findings support the idea that current GDMT for HFrEF may offer prognostic benefits in patients with an LVEF > 40% whilst reiterating the need to recognize the somewhat arbitrary nature of LVEF cut-offs, an appreciation of the intra-operator variability in measuring LVEF [11], and perhaps a move to treating “borderline” LVEF HF cases in the same way as HFrEF patients from a management perspective.

## 3. Sodium–Glucose Cotransporter-2 (SGLT2) Inhibitors

SGLT2 inhibitors are recommended as part of the first-line treatment for heart failure with reduced ejection fraction (HFrEF), heart failure with mildly reduced ejection fraction (HFmrEF) and heart failure with preserved ejection fraction (HFpEF) by the ESC [9]. The EMPEROR-Preserved trial demonstrated that the combined risk of CV death or HF hospitalization for patients with HFpEF was reduced with Empagliflozin vs. placebo and, crucially, these findings extended to those patients with or without T2DM [15]. The PRESERVED-HF trial was the first to demonstrate an improvement in patient-reported symptoms and exercise tolerance when using Dapagliflozin vs. placebo in HFpEF patients [16]. These trials are visually represented in Table 1. Additionally, SGLT2 inhibitors have added benefits in patients with chronic kidney disease (CKD) by helping to prevent progression [17], which is a common co-morbidity of HF patients, as well as adverse effects from other guideline-directed heart failure therapies. This class of medication was initially developed to treat T2DM and has found its place amongst GDMT for HF, an additional benefit discovered owing to a progressive understanding of the interplay between CVD-associated co-morbidities.

For example, patients with diabetes are more than twice as likely to develop HF [6]; the correlation between increasing weight and HFpEF is well established, and CKD disproportionately affects the elderly population and those with underlying CVD [18]. In addition to this, HFpEF patients have lacked any evidence-based prognostic treatment options for many years.

**Figure 2:**
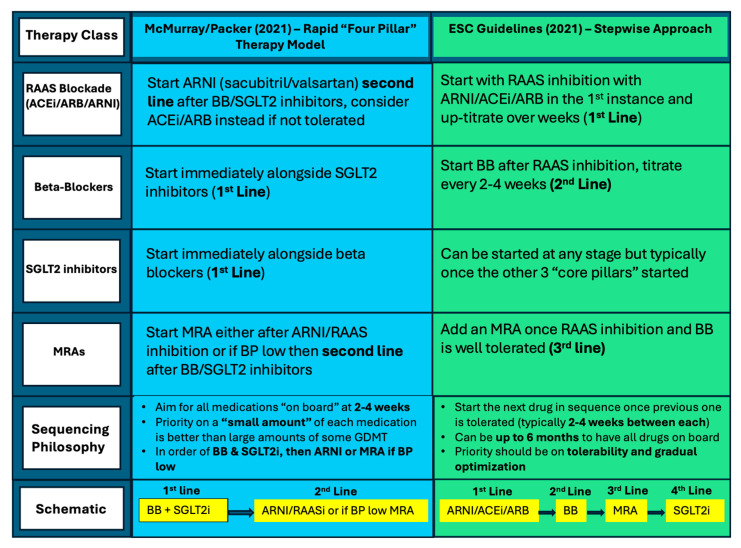
Visual schematic comparing the different proposed sequencing strategies for patients with heart failure with reduced ejection fraction (HFrEF) as described by the ESC Guidelines (2021) vs the McMurray/Packer (2021) approach for different medications within GDMT [9,19].

Medical therapy up-titration in HF is a good example of when treating one organ system can often adversely affect another. Some of the most common reasons for up-titration failure involve side effects from drug classes affecting renal function, potassium levels and symptomatic hypotension. In contrast, SGLT2 inhibitors can be used in CKD and are renally protective; existing in one standard dose and in tablet form means no up-titration and therefore better compliance [17]. These benefits are supported by more recent suggestions of how to sequence the initiation of HF medications in patients, with one current proposal focusing on getting four drugs “on board” within four weeks and starting both an SGLT2 inhibitor and a beta-blocker in the first instance, as seen in comparison to the ESC Guidelines sequencing approach in Figure 2 [19]. It is well established that each of the “four pillars” of HF medication offer reductions in both morbidity and mortality within 30 days of starting treatment [20]. Therefore, prioritizing at least “small doses” of each drug rather than maximal up-titration of one before initiation of another should improve patient outcomes. SGLT2 inhibitors are generally well tolerated, and beta blockers have arguably the most significant individual benefit for HFrEF patients, which supports this therapeutic approach.

Beyond the established benefits of SGLT2 inhibitors in HF, there is emerging evidence that this medication class exhibits pleiotropic effects with benefits for patients with other cardiovascular conditions, including valvular heart disease, IHD, arrythmias, and cardiomyopathies, and in cardio-oncology patients [21]. Recent evidence has shown that patients with HFrEF with an implantable cardioverter defibrillator (ICD) or cardiac resynchronization therapy defibrillator (CRT-D) given an SGLT2 inhibitor over a 1-year follow-up had significantly reduced atrial and ventricular arrythmia events post initiation [22]. Moreover, these wider benefits described apply to populations with or without diabetes, which may open new avenues for their prescribing reach beyond HF, CKD and T2DM in the future [21].

Ultimately, SGLT2 inhibitors are easy to initiate in the hospital setting, can be started safely during an episode of HF decompensation, and have demonstrable multi-organ benefits. Despite this, side effects including the possibility of increased risk of thrush due to glycosuria and risk of volume depletion owing to its diuretic effect may disproportionately affect the elderly. Furthermore, for patients with T2DM on insulin or sulphonylureas, specialist initiation is recommended due to risk of hypoglycemia, possibly limiting some community prescribing. Overall, they have become first-line options for a majority of high-risk CVD patients, having been the first treatment that showed a reduction in all-cause mortality across the entire LVEF range of HF patients [23], and will likely remain a major part of HF GDMT in the future.

## 4. Finerenone—A New Treatment Option for HFpEF and CVD Patients

Similarly, Finerenone has been investigated in a pooled analysis of three randomized controlled trials (FINE-HEART) with the patient group having CKD, T2DM and heart failure, as it is becoming increasingly clear that the combination of these co-morbidities tends to exist in patients with CVD [24]. Despite this analysis not demonstrating a statistically significant reduction in cardiovascular death, it did demonstrate that all-cause death, numbers of MACEs and HF hospitalizations were significantly reduced when compared to placebo [24]. Additionally, there was strong evidence of renal protection in the kidney composite endpoint (sustained reduction in eGFR > 50% baseline, kidney failure or death from kidney failure) [24]. Recent data has demonstrated that Finerenone, unlike other steroidal MRAs (Spironolactone or Eplerenone), when given to patients with an LVEF of 40% or greater reduced the risk of total HF events and causes of death related to CVD when compared to placebo [8]. Moreover, traditional hormonal side effects of the steroidal MRA alternatives like gynecomastia and other systemic hormonal issues are avoided when using Finerenone and it is associated with a lower risk of hyperkalemia when compared to them [25].

From a therapeutic perspective, these data represent a potential breakthrough for patients with heart failure with preserved ejection fraction (HFpEF) who have traditionally had limited medical treatment options, and we might expect Finerenone to form a part of the future evidence-based therapeutic approach in these patients. Despite this data, Finerenone has not yet been approved by NICE in the UK in the treatment of HFpEF, but new guidelines are currently in progress [26]. Furthermore, whilst the pooled FINE-HEART analysis findings were derived from RCTs, the subgroup populations of each were different, and certain important metrics were excluded because of a lack of inclusion across all trials, for example, urgent HF visits [24]. Further details of the FINE-HEART analysis can be found visually represented in Table 1. There are multiple future studies on the horizon that are aiming to cement the status of Finerenone as part of GDMT for HF patients and, crucially, this includes patients with a spectrum of LVEF [27].

The REDEFINE-HF trial is due to be completed in 2026 and will assess the safety and tolerability of Finerenone in hospitalized patients with an acute decompensation episode, including HFmrEF and HFpEF patients [28]. This should help to extend the future prescribing remit of Finerenone beyond those with stable HF.

The FINALITY-HF trial, due to be completed in 2028, will specifically study HFrEF patients who are intolerant of standard, steroidal-based MRAs and assess whether Finerenone offers a similar prognostic benefit as alternative medication [29]. This could mark a landmark shift in GDMT if the benefits offered by Finerenone are in keeping with traditional MRAs, but with a better safety profile, marking it as a possible future first-line treatment option.

Finally, the CONFIRMATION-HF trial, due to be published in 2028, aims to study the effects of the combination of empagliflozin with Finerenone in patients with HF irrespective of LVEF vs. standard care on the primary outcomes of time to all-cause mortality, total HF events and the complete timeline of these events [30]. Therefore, if positive synergistic patient outcomes are demonstrated, then this could further establish Finerenone within GDMT, as the benefits of SGLT2 inhibitors individually, across the range of HFrEF, HFmrEF and HFpEF patients, are already known [23].

Overall, Finerenone may soon be in the prescribing arsenal for clinicians internationally for HFpEF patients, alongside SGLT2 inhibitors and, possibly, as discussed further, GLP-1RAs in the years to come.

## 5. Glucagon-like Peptide One Receptor Agonists (GLP-1RAs)

The current most widely discussed addition to the line-up in terms of new medication classes are the GLP-1RAs. These are currently licensed for use in T2DM patients and those with a high body mass index of greater than 35 for weight loss or those with a BMI of 30–35 who meet criteria for referral to a specialist weight management service in the United Kingdom (UK) [31]. In recent years, due to the surging rates of T2DM and its direct correlation with worse CVD outcomes, several cardiovascular outcome trials were undertaken, which consistently showed reduced risks of MACEs in patients with T2DM when taking these agents [32]. In 2023, the “Semaglutide and Cardiovascular Outcomes in Obesity without Diabetes” (SELECT) trial was the first dedicated randomized controlled trial (RCT) showing that weekly subcutaneous semaglutide provided a 20% reduced risk of non-fatal myocardial infarction, stroke, and death from CVD in overweight and obese patients without diabetes when compared to placebo [33]. More importantly, post hoc analysis of the data discovered that these CVD benefits were maintained in the long term for patients that lost at least 10% of their body weight [34]. This realization supported the decision for recent additional new licensing in the USA by the Food and Drug Administration (FDA) to recommend prescribing semaglutide as a primary prevention to reduce the risk of CVD outcomes in patients classed as overweight or obese [35]. However, it remains important to appreciate that post hoc analyses have an increased risk of false positives, and as the SELECT trial was not a primary prevention trial its results cannot be applied to all individuals in the prevention of adverse CV events [34]. The above suggests that NICE may need to review their own guidelines on semaglutide use, as currently having a weight loss reduction of less than 5% after a period of 6 months is a recommendation to discontinue treatment [31].

The STEP-HFpEF trial assessed the use of once-weekly semaglutide vs. placebo in patients with heart failure with preserved ejection fraction (HFpEF) and obesity but without T2DM on patient-reported symptoms and weight loss with 1 year of follow-up [36]. The trial confirmed that significant weight loss was maintained in those continuing semaglutide and that self-reported symptoms and exercise tolerance improved vs. the control group [36]. These results were mirrored in the STEP-HFpEF DM trial that had similar primary outcomes but with a patient cohort known to have T2DM [37]. A particularly interesting aspect of the outcome of this trial was that despite the weight loss with semaglutide being, on average, 40% less in patients when compared to the STEP-HFpEF trial patient cohort, the observed benefits for self-reported HF symptoms were broadly similar [37]. This could imply that the mechanistic benefits of semaglutide on the pathophysiology of heart failure extend beyond the weight loss element that was thought to be the primary contributor.

It is becoming clear that GLP-1RAs offer CVD prognostic benefits in their own right, separate to their solely intended function of tackling weight loss. However, despite these favorable results, smaller trial cohorts (LIVE and FIGHT) involving other GLP-1RAs such as Liraglutide and in patients with heart failure with reduced ejection fraction (HFrEF) have shown trends without statistical significance of worsening CV outcomes [32], suggesting that larger-scale trials are needed before widespread rollout. The LIVE trial demonstrated no significant changes in LVEF when compared to placebo and reported higher rates of serious cardiac events (arrythmias, aggravation of known ischemic heart disease, HF and death), and elevations in heart rate were associated with taking this medication [38]. The FIGHT trial similarly showed no significant difference between placebo and study population groups in its primary endpoint, which reflected a global ranked score based on HF rehospitalization event times, proportional N-terminal pro-B-type natriuretic peptide and time to death [39]. Despite these small cohorts demonstrating no significant difference vs. placebo in primary CV outcomes, the LEADER trial with a population of 9340 patients looked at CV benefit when added to standard of care for T2DM patients and demonstrated that liraglutide had a significantly lower risk of the primary composite outcome vs. placebo [40]. This primary outcome with significance included first occurrence of CV death and all-cause death and showed trends without significance of a reduction in non-fatal myocardial infarction and non-fatal stroke [40].

Moreover, Dulaglutide was studied in the prospective REWIND trial that looked at MACEs vs. placebo for individuals with T2DM, either with or without CVD, when added to existing antihyperglycemic therapy regimens [41]. The trial demonstrated trends without statistical significance of decreased CV risk for outcomes of CV death and non-fatal myocardial infarction but did demonstrate significance for non-fatal stroke [41]. This trial differed from other GLP-1RA trials for Dulaglutide as it assumed superiority to placebo rather than non-inferiority, had a majority of patients without established CV disease compared to other trials and had an extended median follow-up of 5.4 years, thus demonstrating its possible benefits for both primary and secondary prevention of CV disease [41].

GLP-1RAs are often referred to in medical literature as their own “group” of medications with generalized outcomes and positive results often attributed in this fashion when it remains clear that agents within this medication class differ between each other and may still need cautionary approaches in their prescribing remit until larger trials and head-to-head CVOTs are completed. Broad comparison of the GLP-1RA trials can be found visually represented in Table 1.

Furthermore, another important consideration in the use of GLP-1RAs in chronic conditions like HF is their side effect profile and how that will affect patient compliance. Multiple studies have shown that serious adverse event rates are low with semaglutide, but it is frequently reported that gastrointestinal (GI) side effects are particularly prevalent [33]. In the SELECT trial, 10% of patients in the semaglutide group vs. 2% in the placebo group reported gastrointestinal symptoms that led to a permanent discontinuation of the medication [33]. In patients with HFpEF who qualify for a GLP-1RA, it is likely that they will need to continue taking the medication lifelong to maintain the weight loss and persistent benefits from improved HF symptoms. This notion is supported by findings in the STEP 1 trial that randomized once-weekly semaglutide or placebo to adults with a BMI of >30 kg/m^2^ for 68 weeks [42]. The trial found statistically significant weight loss in the semaglutide group [42]. Interestingly, this was then followed by the STEP 1 trial extension that looked at a representative subset of these patients that had discontinued treatment and assessed weight change with one year of follow-up, showing that patients regained two-thirds of their prior weight loss [43]. It is clear that sustained weight loss is linked to ongoing medication adherence, but adverse GI side effects may limit this possibility for many patients.

Since their conception, GLP-1RAs have existed in subcutaneous form, which can additionally act as a barrier to uptake. In 2019, Semaglutide received FDA approval for use as an oral tablet to be taken once daily and has been investigated as part of the “Semaglutide cardiOvascular oUtcomes trial” (SOUL) trial for its effect on CVD outcomes in patients with diabetes [44]. Results of this trial have shown a 14% reduction in risk of MACEs in adults with T2DM when taking an oral form of Semaglutide compared to placebo [45]. Moreover, the “Oral semaglutide 50mg taken once per day in adults with overweight or obesity” (OASIS 1) trial showed that semaglutide in addition to lifestyle factors versus placebo produced meaningful weight loss in patients without diabetes [46].

These outcomes may pave the way for this drug class to be used in primary care for primary prevention of CVD risk, as once-daily tablet forms will likely lead to a revolution in the prescribing reach and accessibility of these agents for patients. With increasing usage, a future encompassing a similar scoring system to QRISK3 for statins based on “big data” for GLP-1RAs could be envisioned where they form part of the standard primary care approach to CVD risk stratification. Further research areas of interest are focusing on the understanding of the precise molecular pathways involved in how insulin resistance leads to CVD, and as more comprehensive “patient-specific” profiling becomes a reality, modern genomic advancements involving single-nucleotide polymorphism analysis may provide information about how these pathways influence CVD risk [47]. In theory, this could lead to a future of “precision medicine” where clinicians are able to tailor medication regimens to each patient and predict their efficacy.

GLP-1RAs have recently become the topic of wider public discourse for reasons of national shortage and ease of access in private healthcare markets [48]. Influencers on social media have marketed these medications as rapid weight loss drugs, creating a private market where demand significantly outweighs supply [49]. The uptake in their prescribing in the private sector has left NHS access limited for some patients [49]. In addition, there are numerous barriers to the implementation of widespread prescribing networks of this medication class, such as physicians themselves not being aware of which patients are eligible, the complexity of treatment algorithms for T2DM management coupled with its current mainstream subcutaneous form and, more widely, a lack of understanding of overall CVD risk and how best to support these patient groups [50]. In the UK, prescription costs are fixed by the NHS unlike other countries with insurance-based models, meaning that patients are unaffected by direct cost. Furthermore, with a possible future shift to greater prescribing of oral-based versions of GLP-1RAs, issues with long-term adherence and initial therapeutic inertia from the patient perspective should continue to improve [50]. This would need to be coupled with appropriate patient education on the long-term use of these medications as it well established that weight loss benefits are lost when stopping the medication and proper patient counseling would be expected at the start of any new prescription.

It is important to recognize that inappropriate marketing of these drugs could be contributing to adverse mental health outcomes and increasing pressure amongst young people in the digital age to take medication as a “quick fix” for perceived body image concerns [51]. Consideration of a regulatory framework for the supply via private pharmacies and the need for comprehensive assessments of patients who have been accessing these drugs without proper consultation need to be at the forefront of future policy decisions. It seems inevitable that GLP-1RAs will become more widely needed as their benefits are shown to apply to larger patient cohorts. These medications could serve as a great tool in combination with others discussed to tackle the modern-day CVD epidemic but, without proper regulation, could also inadvertently have some unwelcome unintended consequences.

**Table 1:** Key clinical trials for SGLT2 inhibitors, GLP-1RAs and the non-steroidal MRA Finerenone with demographics on study population and main cardiovascular outcomes highlighted.

Trial	Drug and Class	Primary Condition	Study Population	Key CV Outcomes
LEADER (2016) [40]	GLP-1RA—Liraglutide	T2DM patients at high cardiovascular risk with HBA1C ≥ 7.0%	9340 patients|LVEF: N/A	Liraglutide reduced the risk of CV outcomes. Significant reduction in death from CV and death from all causes compared to placebo.
FIGHT (2016) [39]	GLP-1RA—Liraglutide	HFrEF post-hospitalization	300 patients with LVEF ≤ 40%|LVEF: ≤40%	No significant difference in CV death or HF rehospitalization; trend toward harm in the liraglutide group.
LIVE (2017) [38]	GLP-1RA—Liraglutide	Chronic HF (HFrEF and HFpEF)	241 patients, LVEF < 45%|LVEF: ≤45%	No significant change in LVEF between groups; increased serious adverse cardiac adverse events observed.
REWIND (2019) [41]	GLP-1RA—Dulaglutide	T2DM patients at high cardiovascular risk with high HBA1C	9901 patients|LVEF: N/A	Dulaglutide reduced the risk of CV outcomes compared to placebo, with significant difference in non-fatal stroke outcome.
SELECT (2023) [33]	GLP-1RA—Semaglutide	Obesity without diabetes	17,604 adults with BMI ≥ 27 and CVD|LVEF: N/A	Semaglutide reduced MACEs by 20%, including significant reductions in CV death and non-fatal MI.
STEP-HFpEF (2023) [36]	GLP-1RA—Semaglutide	HFpEF with obesity	529 patients with LVEF ≥ 45%|LVEF: ≥45%	Semaglutide improved KCCQ scores and reduced body weight by 13.3% vs. 2.6% (placebo).
OASIS 1 (2023) [46]	GLP-1RA—Semaglutide (Oral)	Obesity	667 adults with overweight/obesity|LVEF: N/A	Semaglutide 50 mg resulted in 15.1% weight loss vs. 2.4% (placebo).
STEP-HFpEF DM (2024) [37]	GLP-1RA—Semaglutide	HFpEF + diabetes	616 patients with LVEF ≥ 45%|LVEF: ≥45%	Semaglutide improved KCCQ and reduced body weight by 9.8% vs. 3.4% (placebo).
SOUL (2025) [45]	GLP-1RA—Semaglutide (Oral)	T2DM with atherosclerotic (AS) CVD, CKD or both	9650 patients with T2DM|LVEF: N/A	Semaglutide reduced MACEs by 14% in T2DM with ASCVD/CKD vs. placebo.
EMPEROR-Preserved (2021) [15]	SGLT2 inhibitor—Empagliflozin	HFpEF	5988 patients with LVEF > 40%|LVEF: >40%	Empagliflozin reduced risk of the composite of CV death or hospitalization by 21%; no significant reduction in CV death or death from other causes.
PRESERVED-HF (2021) [16]	SGLT2 inhibitor—Dapagliflozin	HFpEF	324 patients with LVEF ≥4 5%|LVEF: ≥55%	Dapagliflozin improved KCCQ scores (symptoms, physical limitations) vs. placebo at 12 weeks.
FINE-HEART (2024) [24]	Non-steroidal MRA—Finerenone	HF + CKD + T2DM	18,991 patients pooled from FIDELIO-DKD + FIGARO-DKD + FINEARTS HF|LVEF: Mixed (mostly preserved)	Finerenone reduced all-cause death, HF hospitalization, MACEs and renal decline in T2DM + CKD.

## 6. Implications for General Medicine When Tackling HF Patients

For primary care physicians, hospital consultants and emergency medicine doctors who may commonly be involved in the care of heart failure (HF) patients, these are some considerations for clinical practice.

HF patients typically have multiple chronic conditions with, hypertension being a commonly associated issue; therefore, stopping a calcium channel blocker or an alpha blocker to mitigate some negative hypotensive effects may give room to continue prognostic HF medications. This also tackles a common issue in the elderly population of polypharmacy and aims to manage blood pressure alongside HF with less medication. Polypharmacy is defined as taking at least five repeat medications, which is typical for a patient with heart failure with reduced ejection fraction (HFrEF) on GDMT and a loop diuretic, with data pooled from the Reasons for Geographic and racial differences in stroke (REGARDS) study showing that 84% of patients on admission for HF and 95% on discharge from an HF admission were on at least five medications [52]. Evidence demonstrates that polypharmacy has an inversely proportional relationship to the initiation of GDMT for HFrEF [53], and strategies such as engaging with clinically advocated de-prescribing tools such as the STOPP/START criteria, using fixed-dose combination (FDC) regimens during the maintenance medication phase and implementing computerized clinical decision systems in hospitals to flag inappropriate therapy will work to counteract these issues [54]. The elderly population are disproportionately affected in their side effect profiles in relation to these agents and, therefore, close renal function monitoring, encouraging patients to monitor their own blood pressure and focused education on self-monitoring of peripheral edema may mitigate many of these side effects.

Frequent HF decompensation admissions are seen following patients with mild acute kidney injury of various cause having prognostic HF medications stopped in the first instance as a blanket approach to minimize progression. Additionally, for those patients that present with decompensated HF, it is important to recognize that a degree of renal venous congestion linked to elevated right-heart pressures can cause kidney dysfunction and would be expected in a proportion of these patients [55]. Therefore, a more cautious approach, especially with SGLT2 inhibitors that can be continued in patients with an eGFR above 20 mL/min/1.73 m^2^ for Empagliflozin and 15 mL/min/1.73 m^2^ for Dapagliflozin, according to the British National Formulary (BNF), may reduce the extent of decompensation [56]. Furthermore, improved prognostic HF medication tolerability and uptake should reduce the need for high levels of loop diuretic use.

It is important to counsel patients on the fact that both MRAs and SGLT2 inhibitors also act as diuretics, information a patient may not be as aware of, and this might lead to better compliance if they are understood to be similar to “water tablets”. HF patients on an MRA may also benefit from a maintenance dose of sodium zirconium cyclosilicate (Lokelma) to allow for continuation if there are associated issues with hyperkalemia [57].

As these newer drugs become more commonplace, we would expect prescribing practices to change and for shared care agreements to be devised for SGLT2 inhibitors, newer MRAs and possibly GLP-1RAs in the future to facilitate community prescribing. This would address an underlying healthcare inequality by bypassing cardiologists in the prescribing of GDMT for HF in the community, as a large majority of these patients are already being managed solely in primary care, and this is especially true for HFpEF patients.

## 7. Conclusions

It comes with great promise that there is substantial evidence to support both an understanding of the mechanistic pathways that link CVD with endocrine and renal diseases and medications that confer benefits for multiple conditions. Creating medications in forms that are easy to continue to prescribe in primary care environments with clearly defined shared care agreements designed by secondary care to facilitate the move to non-specialist prescribing in the community will allow larger patient cohorts to reap prognostic rewards whilst achieving a stranglehold on the worsening CVD epidemic. Now that these medications are becoming more readily available and making their way into international guidelines, there should be a focused push for NICE to approve them in the UK where evidence supports their usage.

## Abbreviations

The following abbreviations are used in this manuscript:

CVD	cardiovascular disease
CKD	chronic kidney disease
T2DM	Type 2 Diabetes Mellitus
SGLT2i	sodium–glucose cotransporter receptor 2 inhibitor
GLP-1RA	glucagon-like peptide 1 receptor agonist
MRA	mineralocorticoid receptor antagonist
HF	heart failure
MACE	major adverse cardiovascular event
CVOT	cardiovascular outcome trial
NICE	National Institute for Health and Care Excellence
eGFR	estimated glomerular filtration rate
